# Supplementary figures and images for: The Relationship between p-tau217, p-tau231, and p-tau205 in the Human Brain Is Affected by the Cellular Environment and Alzheimer’s Disease Pathology
Source: Cells. 2024 Feb 11;13(4):331. doi: 10.3390/cells13040331 (PMC10887205; doi:10.3390/cells13040331)

Cohort II

ITG

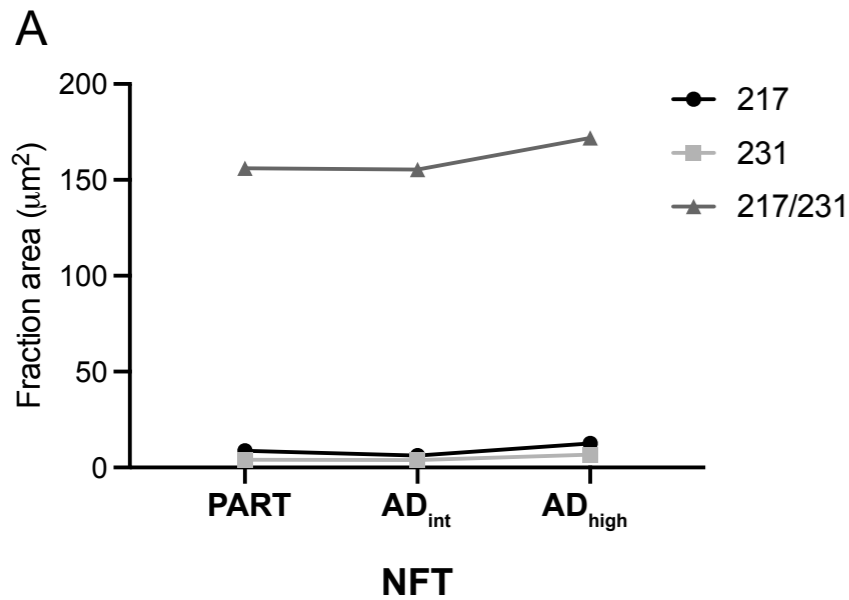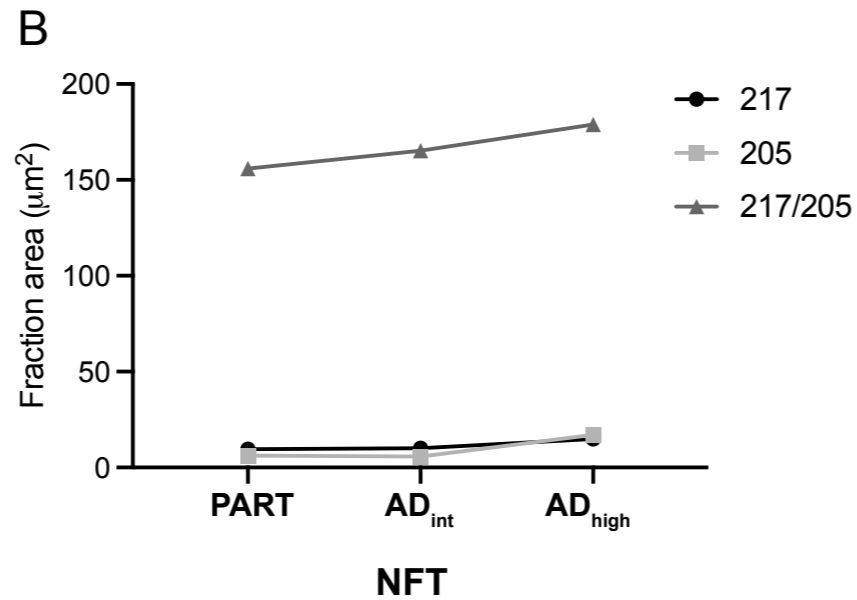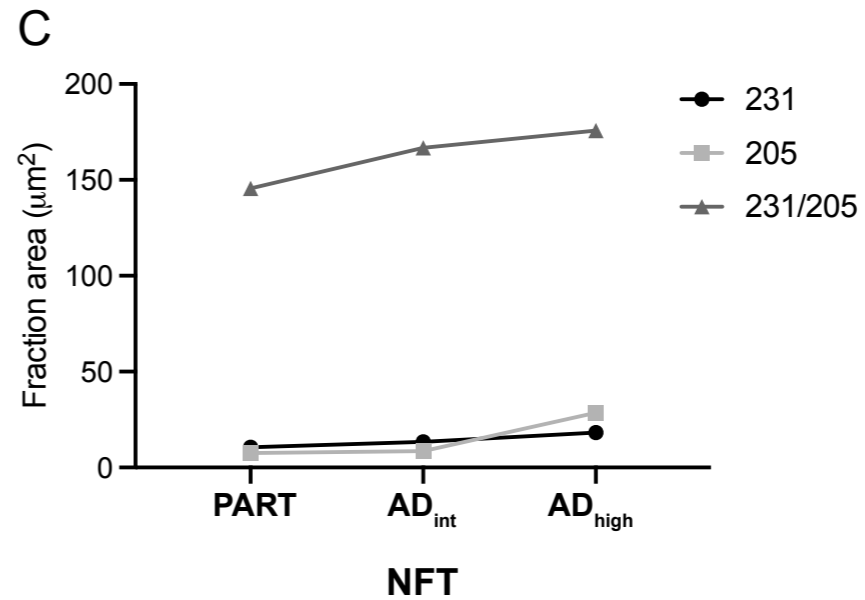

Supplement: Supplementary file 1 [file cells-13-00331-s001.zip › cells-2823039-supplementary.pdf]
